# TUDCA as an autophagic modulator of ATTR V30M Amyloidosis

**DOI:** 10.1186/1750-1172-10-S1-P10

**Published:** 2015-11-02

**Authors:** Cristina Teixeira, Maria João Saraiva

**Affiliations:** 1 I3S - Instituto de Investigação e Inovação em Saúde, Universidade do Porto; IBMC - Instituto de Biologia Molecular e Celular, Molecular Neurobiology, 4150-180, Porto, Portugal

## Background

Different compounds have been studied for the treatment and/or symptoms amelioration of Familial Amyloidotic Polyneuropathy (FAP). We previously showed that Tauroursodeoxycholic acid (TUDCA), a biliary acid with anti-oxidant properties known to reduce non-fibrillar TTR aggregates is also capable of diminishing ER stress by its action on Bip in young transgenic Val30Met mice. This compound has already proved to be neuroprotective in several studies using genetic animal models of Huntington's and Parkinson's disease although the mechanisms underlying its neuroprotection action are still unknown.

Other cellular mechanisms are being explored for possible actions of TUDCA, with special highlight to autophagy, a cellular mechanism that involves the delivery of large protein aggregates, defective organelles and other cellular debris to lysosomes for degradation that has lately been linked to several degenerative diseases where it appears to be impaired.

The objective of our work is to investigate whether improvements previously observed in TUDCA-treated Val30Met mice involves autophagy.

## Methods

For this study, nine months old mice bearing the human TTR Val30Met mutation, in a TTR null background-hTTR Met30 were used. Animals were treated with TUDCA (500 mg/kg/day) in the drinking water, for a 3 month period after which the animals were sacrificed and organs from gastrointestinal tract were collected. An age-matched control group of animals was maintained in the same conditions, drinking regular tap water.

Immunohistochemistry analyses were performed in order to evaluate the expression levels of an autophagic marker, p62, a protein naturally degraded in the final steps of the autophagic flux and that typically accumulates when autophagy is impaired.

## Results

Our preliminary results point to a significant reduction in p62 accumulation in colon samples of TUDCA treated mice.

## Conclusion

TUDCA is a promising compound that has already been proved to significantly reduce TTR toxic aggregates in vivo which points to its capability of modulating TTR aggregation by cellular responses, such as by oxidative stress, ER stress and apoptosis. Our results indicate that this modulation involves the autophagic machinery where it seems to enhance/restablish the autophagic flux that in turn may be directly involved in the pathological cascade in FAP, thus TUDCA contributes to the clearance of Val30Met TTR aggregates.

